# Clinical Review of Neuromusculoskeletal Complementary and Alternative Approaches for the Treatment of Chronic Pelvic Pain Syndrome

**DOI:** 10.7759/cureus.27077

**Published:** 2022-07-20

**Authors:** Stephanie K Marks, Nathan A Rodriguez, Anisha Shah, Andi N Garcia, Leah Ritter, Angela N Pierce

**Affiliations:** 1 Curriculum & Integrated Learning, College of Osteopathic Medicine, Kansas City University, Kansas City, USA; 2 Curriculum & Integrated Learning, College of Osteopathic Medicine, Kansas City University, Joplin, USA

**Keywords:** myofascial release, acupuncture, manipulation, complementary and alternative medicine, pain, chronic pelvic pain syndrome, chronic prostatitis

## Abstract

Chronic pelvic pain syndrome (CPPS) is a functional pain disorder characterized by ongoing pain in the apparent absence of clinically identifiable causes. The prevalence of functional pain disorders demonstrates the importance of adequate management of ongoing symptomatology, but due to the uncertain etiology and myriad patient presentation phenotypes, reliable treatment options are difficult to implement. New interventions involving non-pharmacological approaches to pain management have been investigated across a spectrum of clinical and pre-clinical studies. Given that conservative care such as exercise, counseling, and musculoskeletal therapy is widely recommended as first-line treatment for CPPS, an updated review of these and related methodologies are needed. Familiarizing physicians and the public with the newest evidence for complementary and alternative medicine (CAM) and other conservative care treatments will assist with the promotion of evidence-based practices in a safe and reliable manner. This review aimed to summarize the current evidence and proposed mechanisms for non-pharmacological treatment specific to CAM and management of chronic pelvic pain centered on neuromusculoskeletal focused intervention such as acupuncture, auriculotherapy, manipulation, manual therapy, myofascial release, and phototherapy. The discussion suggests that reported improvements in pelvic pain or related symptomatology may be attributed to changes in the peripheral inflammasome and somatic origins of peripheral sensitization. Robustness of the included clinical studies is discussed throughout the review, and attention is paid to delineating inclusion criteria of formally diagnosed CPPS compared to general pelvic or abdominal pain. Overall, this review consolidates the current state of evidence regarding the utilization of non-traditional interventions using CAM techniques for the management of chronic pelvic pain and recommends a future direction for the field.

## Introduction and background

Chronic pelvic pain syndrome (CPPS) and chronic prostatitis/chronic pelvic pain syndrome (CP/CPPS) are conditions acknowledged worldwide, affecting up to 16% of females and 10% of males, respectively [1-8]. Despite the global prevalence, the terminology associated with chronic pelvic pain remains inconsistent in the literature. This is likely due to the nebulous presentation of symptoms and necessity of exclusion criteria required to formulate a diagnosis. As diagnostic nomenclature, CPPS is typically reserved for those with reported pain experienced during at least three of the previous six months, lacking in apparent pathognomonic causes, and is unrelated to reproductive or hormonal cycles. Pain can be generally diffused throughout or quite localized within the pelvis, which is anatomically delineated as the area inferior to the umbilicus and superior to the symphysis pubis. Although the inclusion of the perineum and reproductive organs varies among the various definitions of pelvic organ anatomy, an array of related disorders affecting pelvic and/or lower abdominal organs can contribute to pain. In females, common diagnoses that contribute to chronic pelvic pain include endometriosis, interstitial cystitis/painful bladder syndrome, and vulvodynia [9,10]. Other disorders such as irritable bowel syndrome, colitis, pelvic floor dysfunction, and neuropathy are shared between males and females. When pain is described as originating from the prostate, testes, and penis, the condition is usually referred to as CP/CPPS. When the source of pain is not readily identified by patients, a centralized pain disorder may be at play.

As a diagnosis of exclusion and given the vast array of presentations and patient history, outcomes from the standard approaches to managing pain are quite variable. The most resistant cases are often emotionally, physically, and financially frustrating for the patient and the physician. Treatment options can deteriorate into a trial and error approach aiming to manage the most deleterious of symptoms that may never resolve. Therefore, investigating additional multi-modal approaches to treating CPPS is urgently needed. The National Institute of Health (NIH) defines complementary and alternative medicine (CAM) as the replacement or supplementation of conventional medicine with non-mainstream approaches [11]. Typically, CAM emphasizes non-pharmacological means to treat the whole person rather than one system or disease. Pain management has been a consistent subject of investigation by integrated medical practices due to the prevalence of therapeutic resistance to chronic pain, the growing awareness of the opioid epidemic, and need for new methods to manage short-term exacerbations and long-term quality of life.

A major limitation of the treatment of CPPS is physician familiarity with this complex, multimodal disorder. In 2009, the results from a query of 289 primary care physicians showed a wide range of familiarity with CPPS [12]. Among the physicians who treated a known or suspected case of CPPS, 72% stated that their most common choice was to treat with antibiotics. The selection of antibiotics as a treatment for CPPS is unsupported by evidence and reserved only for suspected cases of infectious, bacterial-based etiologies. Unsurprisingly, CAM treatments were rarely recommended. This is in contrast to national recommendations by societies representing the United Kingdom [13], Japan [14], the United States [15], and Canada [16], which recommend conservative treatment as the first-line approach for CPPS, including addressing stress relief, dietary modification, exercise, musculoskeletal physical therapy, and psychological support. Interestingly, despite recommendations of national societies, a recent review concluded that China was the largest contributor to randomized controlled trials investigating CAM interventions for health conditions in the last decade [17]. This highlights the importance of bringing awareness of CAM-focused studies across the globe, particularly when clinical studies investigate treatments of a commonly resistant condition such as CPPS. There remain systematic challenges to implementing CAM-based clinical studies, which are often reflected in the small to moderate-sized treatment groups and lack of robust controls.

The purpose of this review was to summarize the CAM approach to the treatment and management of CPPS and include methods less frequently reviewed elsewhere. Each section describes a current snapshot of the clinical atmosphere surrounding the approach as well as a discussion on clinical or preclinical insight into proposed mechanisms underlying any demonstrable efficacy. We discuss the quality and scope of evidence-based studies and the areas of continued. Ultimately, the review updates the current state of knowledge related to clinical relevance, proposed mechanisms, and discussion pertinent to the management of this difficult condition.

## Review

Methods

The online biomedical journal databases PubMed and EBSCOhost were queried between April 1, 2021, and August 1, 2021. Searches were carried out using the following combinations of keywords and Boolean operators (“AND,” “OR”): ({chronic pelvic pain syndrome} OR {CPPS} OR {chronic prostatitis chronic pelvic pain syndrome} OR {CP/CPPS} OR {pelvic pain}) AND ({complementary and alternative management} OR {CAM} OR {myofascial release} OR {manual therapy} OR {manipulation} OR {acupuncture} OR {electromyography}). Results were indexed and duplicates were removed. Articles were reviewed if the full text was available in English. Otherwise, non-English articles were evaluated if there was an English-available abstract containing sufficient information. Any non-English full-text publications referenced in the final review are denoted as such. Only human studies designed in the prospective, retrospective, or randomized clinical trials were included. Case studies were excluded. Records were grouped according to the type of intervention and analyzed for inclusion criteria, number of subjects enrolled, and outcome measurements and presented in the following narrative review.

Manipulative therapy

In 1989, Browning published a study that demonstrated improvement in low back pain among six patients that met the criteria for a generic pelvic pain disorder following sacral nerve root decompression [18]. This study was not randomized among participants, focused only on immediate effects, and captured minimal outcome measures, but it did become the inspiration for a more robust albeit pilot study of manipulative therapy for improving CPPS-related pain that is described next. Hawk et al. published a prospective study in 1997 that demonstrated improvements in reported pain and disability scales in patients with chronic pelvic pain following six weeks of lumbosacral manipulation and trigger point treatment compared to baseline [19]. Although this study did not follow the patients long-term nor use a randomization protocol, it was quite novel for its time. Since then, the field has recognized that recruitment of formally diagnosed CPPS patients into true randomization and administration of sham treatments for manipulation techniques are difficult to execute [20]. Subsequent studies have become more elegant as a result. More recently, there has been development in the treatment of idiopathic pain conditions, functional pain disorders, and chronic visceral pain of varying systems involvement using one of several manipulation and manual techniques that are described next.

Osteopathic manipulative therapy (OMT) focusing on pelvic girdle biomechanics, abnormal tension and somatic dysfunction at the pubic symphysis has been suggested as a potential intervention for CPPS [21]. A clinical trial conducted in Germany showed improved symptom scores and quality of life in patients with CPPS [22]. While the full article is published in German, several follow-up studies have followed since then that suggest a long-term benefit of OMT for this patient population [23]. A study from China with 60 patients with functional abdominal pain syndrome reported pain and other generic abdominal symptoms either markedly or completely resolved following spinal manipulative treatment [24]. These findings were replicated in a follow-up study by the same researchers [25]. While not specifically recruiting patients with CPPS, the inclusion criteria for the enrolled participants describe ongoing pain affecting the general abdominal region with many symptoms indistinguishable from CPPS. Importantly, the participants described visceral pain concurrent with back pain and that spinal manipulation of the thoracolumbar vertebrae improved symptoms of both. The vague definitions that distinguish pelvic from abdominal pain are important terms that should be required as reporting elements for clinical enrollment for study purposes, but due to the non-distinct borders of pain symptoms, confusion among practitioners worldwide, and fluctuating criteria, mandatory exclusions may be impractical.

Myofascial release

Another key area where manipulative therapy providers receive training is advanced anatomical understanding of the human body and the ability to palpate impaired or altered muscular, fascial, skeletal, or lymphatic mechanics. These altered systems are commonly referred to as somatic dysfunctions and arise due to homeostatic imbalances [26]. Somatic dysfunction manifests in palpatory findings that are otherwise undetectable through radiographic or laboratory exams. Moreover, through training and clinical practice, manipulation specialists tend to perform musculoskeletal and neurological physical exams that also assess posture, gait, lumbar, sacral, and innominate mechanics, as well as palpation of the fascia, ligaments, and muscles that surround these regions. Specifically, the pelvic diaphragm (or levator ani) which makes up the pelvic floor and inserts on the symphysis pubis is a common site of treatment for myofascial release among CPPS patients.

Manual therapy targeting myofascial trigger points in the pelvic floor for interstitial cystitis and urgency frequency syndrome, which are common components of CPPS showed promising results [27]. The study enrolled 42 patients who had urgency frequency syndrome with or without pain and of which 10 were formally diagnosed with interstitial cystitis. Interestingly, the majority (86%) of participants were female, which is important because females have been shown to exhibit greater number and intensity of trigger points compared to males with CPPS [28]. Respectively, the two groups had an average of approximately one to two visits per week over the duration of two to three months. Of the patients diagnosed with urgency-frequency syndrome, 84% had moderate, marked, or complete resolution of symptoms, and long-term follow-up (20 months) showed 73% of patients had a sustained improvement. Furthermore, all 10 of the interstitial cystitis patients showed some form of improvement, and the majority (70%) demonstrated significant reduction in pain and voiding symptoms. In another study of 374 patients that looked at myofascial trigger points and CPPS, medication usage decreased following treatment [29]. At baseline, 63.6% of patients took at least one medication related to CPPS management. Following one month of treatment, the percentage of patients taking medication was reduced to 44.8%, and after six months of treatment, the percentage decreased slightly to 40%. Another clinical trial demonstrated a more broad benefit of treating myofascial trigger points in the pelvic floor for patients with CPPS. The trial found that myofascial therapy improved hypersensitivity responses to thermal and mechanical pain [30], suggesting an improvement in overall pain thresholds. This is consistent with other work that support peripheral hypersensitivity exists among CPPS patients [31].

These findings suggest some degree of pelvic peripheral sensitization and the neuromodulation therapies that seek to diagnose and ultimately treat these changes have been consolidated into a field of study preliminarily termed neuropelveology, a discipline seeking to gain traction among mainstream pain researchers and clinicians [32]. While the focus of these neuropelveology studies is highly characterized by investigations into mechanisms contributing to peripheral neuropathies, central changes in pain processing cannot be overlooked. The wide variety of disparate etiologies thought to contribute to CPPS speaks to the range of conditions that may all manifest with similar patient presentations and ultimately lead to CPPS syndromes. Therefore, patient history and careful review of symptoms, triggers, and exacerbation patterns must be taken into account when searching for appropriate management options individualized to the patient’s primary need [30].

A multimodal musculoskeletal approach to treating general pelvic floor dysfunction, including internal myofascial release, physical therapy, biofeedback, and strengthening exercises improved urinary and pain symptoms among males with CPPS [33]. Similar robust symptom improvements were retrospectively identified among males with CPPS following external myofascial release [34]. Myofascial release improved reported pain in a longitudinal study of women with CPPS up to nine months post-treatment, and also increased the anatomical width of the levator ani muscle, suggesting improvement in adhesion-induced muscle contraction or shortening [35]. While these molecular outcomes suggest a mechanism by which reported pain is reduced when patients are treated with myofascial release, they do not fully reveal the cellular mechanisms involved when palpating fascial restrictions and resolution [36,37]. Clearly, evidence-based research needs to continue to be collected to demonstrate safety, tolerance, duration, and effectiveness of varying forms of manipulative therapy as treatment for CPPS.

Electromyography biofeedback and electromagnetic therapy

Biofeedback that incorporates electromyography (EMG) instrumentation is a neuromodulation technique that combines two concepts. The first is biofeedback, which educates the patient about their condition for the purposes of increasing self-awareness and control. The second concept is the visual or audible mapping of neuromuscular electrical activity. Upon interpretation by a skilled practitioner, the electrical activity is scored and analyzed for proper levels similar to the interpretation of any electrophysiological technique. Dysfunction may be due to pathological firing patterns, but the most common finding is quantified signal intensity reflecting a presumed level of hyper- or hypotonic muscle tension [38]. Then the patient can intentionally retrain the muscles into an optimal firing pattern using a biofeedback mechanism. This can be done while the patient is connected to the EMG, in which the practitioner can set goals for the appropriate amount of electric activity a muscle requires for retraining. Surface EMG (sEMG), which adheres the electrodes directly to the surface of the skin superficial to the targeted muscle uses a similar mechanism and has been used to improve symptoms of both chronic low back pain [39,40] and chronic neck pain [41].

The utilization of EMG techniques to improve outcomes related to musculoskeletal pain is a logical treatment modality. Less commonly known, EMG biofeedback training has also been used for pelvic floor rehabilitation particularly targeting dysfunction in the pelvic diaphragm, levator ani, pubococcygeus, puborectalis, iliococcygeus, and the obturator internus muscle [42]. For the purposes of treating muscular involvement of pain associated with CPPS, EMG biofeedback utilizes intravaginal, transperineal, or transrectal placement of the EMG probe. With respect to CPPS in females, there have been a few studies depicting EMG biofeedback improves pelvic pain in general [43,44]. In a more recent study from 2017, EMG biofeedback combined with electromagnetic stimulation improved reported levels of pelvic pain and dyspareunia compared to electromagnetic stimulation alone [45]. Electromagnetic therapy significantly improved pain-related symptoms in males with CPPS in a randomized controlled trial against placebo therapy [46]. Adding biofeedback to electromagnetic stimulation of pelvic floor muscles significantly improved quality of life and reported pain and urinary symptoms in males with CPPS compared to stimulation alone; although, both groups benefitted from the EMG intervention compared to baseline. It is important to note that the biofeedback component of EMG and electromagnetic stimulation appears to be a critical element of intervention.

The therapeutic effect by which EMG biofeedback exerts beneficial benefit is not known, but three mechanisms may be derived from the literature on sacral nerve stimulation for the treatment of urinary symptoms associated with pelvic pain [47]. They include stimulation of descending pain inhibition pathways at the level of the dorsal horn, restoration of normal signal connectivity between the brainstem and cortex, and interruption of the efferent firing pattern in pelvic floor muscles to break the cycle of muscle spasm [46,48].

Photobiomodulation therapy

Low-level laser therapy (LLLT), also known as cold laser and photobiomodulation therapy, is a light source treatment that is thought to act via photochemical or non-thermal reactions in mammalian cells. Cold laser therapy differentiates itself from hot lasers by its power output. Hot lasers have power output above 500 milliwatts whereas cold lasers have a power output at or below 500 milliwatts [49]. Cold laser therapy is also non-thermal whereas hot laser therapy produces heat. LLLT focuses on irradiation over the skin at specific traditional Chinese medicine acupuncture points or at muscular trigger points [50]. Although no identified studies have assessed the effectiveness on CPPS, LLLT has been shown to improve syndromes such as low back pain [50], neck pain [51], painful diabetic neuropathy [52], and pelvic pain associated with primary dysmenorrhea [53,54].

Extracorporeal shock wave therapy (ESWT) is distinct subtype of low-level phototherapy that utilizes many of the same principles through the introduction of a pressure wave of short duration (approximately 10 μs) at a frequency of 16-20 MHz [55]. The low-energy version of ESWT pulses 200-300 shocks per treatment. The non-linear pressure waves generate cavitation that physically perturbs internal tissues and has been shown to impact cell membrane electrophysiology, reactive oxygenation species formation, cell proliferation, and cell growth with particular impact on the extracellular matrix [56].

Human studies of CPPS patients have demonstrated improved quality of life, pain reports, and urinary symptoms following ESWT therapy. Two recent meta-analyses [57,58] have previously reviewed these clinical trials and will not be repeated here, but in brief, the research is promising. In fact, a very recent 2021 randomized controlled trial showed acute decreases in reported pain, quality of life, and urinary symptoms in males with CPPS that persisted up to four-week post-treatment [59]. Given the minimal reported side effects and minimal invasiveness of ESWT, photobiomodulation therapy should be considered potential adjunctive treatment for patients with CPPS. The primary caveat to note is that the majority of the studies focus on males with CPPS. Among studies featuring females with CPPS, the ESWT intervention primarily monitors symptoms associated with dysmenorrhea or related cyclical pain, which may confuse findings given the wide acceptance that CPPS-associated pain is unrelated to reproductive cycles.

Acupuncture 

Acupuncture is thought to have originated in China around approximately 2500 BC. Not surprisingly, the therapeutic mechanism of acupuncture was heavily influenced by culture during that era [60]. Acupuncture is the practice of penetrating the skin with thin needles which are then activated through gentle, specific movements of the practitioner's hands or via electrical stimulation (electrical acupuncture) [60]. Acupuncture has been used to alleviate pain associated with cancer treatments [61] as well as low back, neck, and joint pain [62-64].

Evidence continues to emerge that acupuncture would be effective for chronic pelvic pain in both males and females. A study in 2015 with 100 patients using a "sham acupuncture" comparison group concluded that the real acupuncture treatment reduced symptoms in males with chronic prostatitis [65]. This study contained 100 patients and was compared to a sham acupuncture group. While total symptomatic score reports decreased in both groups at six weeks, the acupuncture group saw a greater therapeutic effect that persisted through 24 weeks. This was reproduced in a similar study in 2018 of 68 patients whereas acupuncture resulted in a greater decrease in reported symptom scores that persisted up to 30 weeks compared to control [66]. Acupuncture also significantly reduced reported pain levels in a group of 55 patients with CPPS, with some patients reporting complete elimination of pain; however, 25.5% reported no benefit and inferior hypogastric block exerted a stronger intervention effect compared to acupuncture [67].

Acupuncture has been shown to alleviate pregnancy-related and endometriosis-related pelvic pain, but specific studies evaluating acupuncture intervention for females with CPPS are lacking [68,69]. A meta-analysis from 2018 identified four randomized controlled trials and concluded that there was a benefit of adding acupuncture to conventional treatment for CPPS in females, but the quality of the study designs was generally interpreted as low [70]. Since then, a therapeutic cupping method targeting a known acupuncture point adjacent to the spinous process of the second lumbar vertebra that is thought to correlate with neurogenic sensitivity of the bladder (acupoint BL23) demonstrated significant improvement in reported pain scale and intensity among females with CPPS [71]. Serum concentration of C-reactive protein decreased from 2.2 ± 0.72 mg/L to 1.32 ± 0.42 mg/L following cupping treatment. Minimal to moderate correlation was identified between reported pain level or pain intensity and the reported change in serum C-reactive protein concentration.

Battlefield acupuncture

Battlefield acupuncture (BFA), a type of auriculotherapy, is a protocol used in the military for rapid pain relief [72]. Despite the reference to combat, BFA is most commonly used by certified providers across many veterans affairs healthcare administration facilities for the treatment of multiple types of chronic pain among veterans [73]. These patient populations contribute to the origin of the majority of BFA research; however, there are potential applications to the use of auriculotherapy with other types of chronic pain patients. The procedure for BFA involves the placement of acupuncture needles in five specific points on the exterior meatus of the ear with the studs remaining in place for up to several days [74].

Two clinical trials suggest that auriculotherapy has a potential benefit for patients with chronic pelvic pain. The first comes from a pediatric gastroenterology practice featuring a randomized clinical trial of adolescent population of patients with functional gastrointestinal disorders lacking a known organic cause. This study demonstrated high levels of tolerance to the therapy and significant decreases in abdominal pain scores following three weeks of treatment. The decreased report of painful events persisted for an extended time period after the treatment concluded [75]. Second, a crossover study demonstrated a non-significant yet trending improvement in reported pain intensity among CPPS patients, specifically [76]. However, the power of this study may benefit from the inclusion of a strict negative control group because the outcomes compared electrical versus manual stimulation of the auricle. It is likely the moderate to minimal effect of the intervention was observed because the control group still induced auricular nerve afferent stimulation, the anatomy of which indicates that the auricular branch of the vagus nerve and the auriculotemporal nerve, which is non-vagal, may still mediate benefit due to the indirect convergence in the brainstem. Beneficial effect, reduced pain medication, and increased patient satisfaction following auriculotherapy have been reported in clinical investigations of a wide variety of chronic syndromes rather than improving discomfort during acute procedural pain. While unrelated to CPPS, it is interesting to note that studies of syndromes such as chronic sore throat [77], migraine [78], neuropathic pain [79], cancer pain [80], osteoarthritis [81], and episiotomy [82] have shown demonstrable benefits from BFA.

An overview of articles included in the review is reported in Table 1, which includes details pertaining to the number of participants from each clinical study. The category of pain by which patients were classified may be specific to CPPS, general pelvic pain, or non-specified functional abdominal pain. The primary outcome measures include a variety of pain-specific scales, quality of life, or associated (non-painful) symptoms. The number of participants (n) varies from six to 374 and the average number of participants was 58. 

**Table 1:** Complementary and alternative medicine supported neuromusculoskeletal techniques for treatment of chronic pelvic pain syndrome CPPS: chronic pelvic pain syndrome; CRP: C-reactive protein; GUPI: genitourinary pain index; IPSS: International Prostate Symptom Score; NIH-CPSI: National Institutes of Health-Chronic Prostatitis Symptom Index; PDI: Pain Disability Index; PFSD: Pain Frequency-Severity-Duration; RAND-36: RAND corporation 36-Item Short Form Health Survey; VAS: visual analog scale

Treatment category	Participants (n)	Pain inclusion criteria	Sex (male, female, both)	Primary outcome measures	Reference
Manipulation	80	Abdominal	Both	VAS pain, symptom improvement scales	[19]
	60	Abdominal	Both	VAS pain, symptom improvement scales	[18]
	35	CPPS	Males	IPSS, NIH-CPSI, quality of life	[16]
	19	CPPS	Males	IPSS, NIH-CPSI, quality of life	[17]
	19	CPPS	Females	PDI, VAS pain, RAND-36 Health Survey	[13]
	6	Pelvic	Females	Pain Likert scales, symptom improvement Likert scales	[12]
Myofascial release	374	CPPS	Both	Symptom improvement scales, medication usage	[23]
	42	Pelvic	Both	Symptom improvement scales	[21]
	39	CPPS	Females	Pain scales, urgency, severity and impact of life questionnaire, levator ani morphology, pain thresholds	[29]
	31	CPPS	Males	NIH-CPSI	[28]
	14	CPPS	Males	NIH-CPSI, GUPI	[27]
Acupoint stimulation	100	CPPS	Males	NIH-CPSI	[59]
	68	CPPS	Males	NIH-CPSI	[60]
	55	CPPS	Females	VAS Pain	[61]
	15	CPPS	Females	Serum CRP, McGill Pain Questionnaire, VAS pain, pelvic pain questionnaire, symptom improvement scales	[65]
BFA	60	Abdominal	Both	PFSD scale, symptom improvement scales	[69]
	15	CPPS	Female	Psychophysical metrics, evoked pain outcomes	[70]
Biofeedback	94	Pelvic	Females	Symptom improvement scales, satisfaction questionnaire	[39]
	33	CPPS	Females	Reported pain, symptom improvement	[37]
	21	CPPS	Males	VAS pain, symptom improvement scales	[40]
Photobiomodulation	30	CPPS	Males	VAS pain, NIH-CPSI	[53]

**Figure 1:**
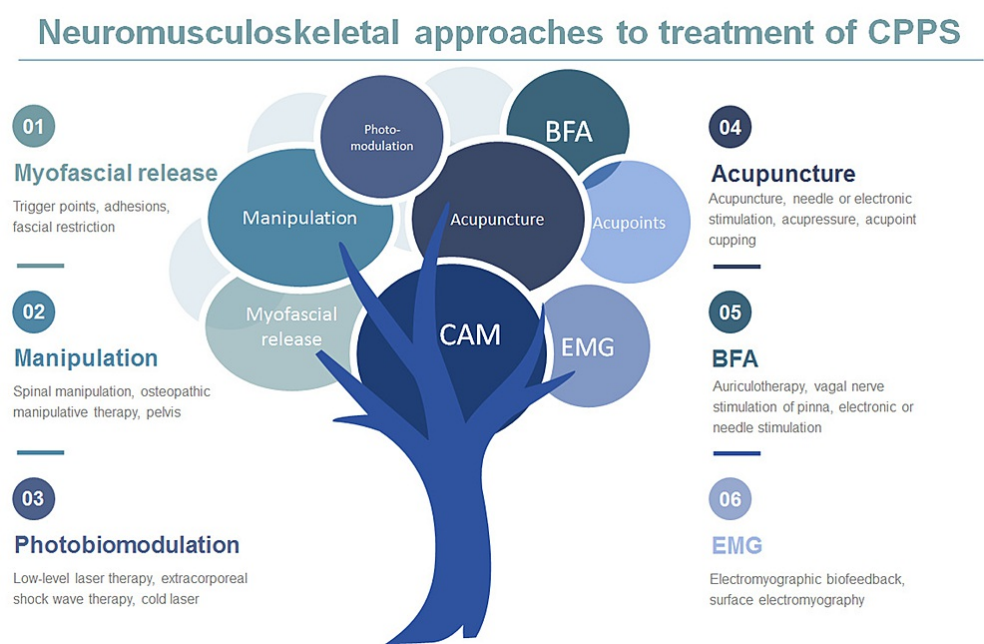
Complementary and alternative (CAM) approaches to treatment of CPPS are collated by neuromusculoskeletal theme. Examples of the approaches are listed with each category. (1) Myofascial release may include specific identification and treatment of muscular trigger points, characterized by adhesions or fascial restriction. (2) Manipulation is another form of manual treatment and includes spinal manipulation, lumbopelvic or lumbosacral manipulation, or osteopathic manipulative therapy. (3) Photobiomodulation therapies include the utilization of various wavelengths of light, specifically using sound waves or lasers at prescribed frequencies. (4) Acupuncture may be classified by needle-penetrating or electronic-stimulatory acupuncture, targeting acupressure points, or acupoint cupping modalities. (5) Battlefield acupuncture (BFA) is a subtype of acupuncture also termed auriculotherapy that specifically targets the pinna of the ear. (6) Electromyographic (EMG) biofeedback includes real-time musculoskeletal contracture states for the purposes of relaxation training or pelvic floor strengthening.

Mechanistic themes with relevant clinical examples that summarize the neuromusculoskeletal approaches in relation to complementary and alternative medicine are shown in Figure 1.

## Conclusions

Adding to the complexity of CPPS is the inclusion of an array of comorbid pain conditions affecting non-pelvic organs. Chronic pain conditions are often comorbid and treating one may or may not improve the symptoms associated with another. First-line treatment of CPPS is the administration of non-steroidal anti-inflammatory drugs and muscle relaxants, but for the complex case, these initial approaches provide inadequate relief. Subsequent management is based on the dominant symptom if one can be identified. For the non-specific or multimodal complaint, neuromodulators may be prescribed in a trial and error fashion that is thought to target an unknown neurotransmitter “imbalance.” Anticonvulsants, tricyclic antidepressants, and serotonin-norepinephrine reuptake inhibitors have shown some benefit for CPPS patients, but there is a wide variation of individual pain responses to these medications. The focus of this paper was to review a variety of neuromusculoskeletal complementary and alternative interventions for the management of CPPS.

Pain management specialists working with treatment-resistant cases of CPPS may consider implementing an alternative treatment either as a stand-alone therapy or in combination with traditional pharmacological options. As many of the complementary and alternative interventions described in this review reported well-tolerability and minimal deleterious effects, their implementation into the overall management plan should at least be taken into consideration. There are some clinical trials or preclinical studies that demonstrate at least minimal benefit to many of the described approaches in this review. However, there will consistently remain a group of patients who are minimally or fully unresponsive to a given intervention. This is likely due to the wide variety of underlying pathologies contributing to likely disparate disorders that are manifesting in a constellation of patient presentation experiences ultimately diagnosed as CPPS. The next era of this research should consider clinical or preclinical trials that combine traditional pharmacological treatments with additional integrated interventions, such as studying anti-inflammatories along with acupuncture, biofeedback techniques that incorporate electromyography instrumentation, and physical therapy, or antidepressants in combination with cognitive behavioral therapy or supplement management and exercise. Bidirectional research needs to take place that combines multimodal approaches to management and simultaneously categorize the CPPS phenotypes either through biomarkers, genetic factors, and relevant histories or triggers. This sort of personalized medicine will someday efficiently connect patients with the combination of interventions that may be best poised to help them.
